# Improving the Conductivity of Solid Polymer Electrolyte by Grain Reforming

**DOI:** 10.1186/s11671-020-03355-4

**Published:** 2020-05-26

**Authors:** Zhaohuan Wei, Yaqi Ren, Minkang Wang, Jijun He, Weirong Huo, Hui Tang

**Affiliations:** 1 School of Physics, University of Electronic Science and Technology of China, Chengdu, 611731 China; 2 Guangdong Provincial Key Laboratory of Energy Materials for Electric Power, Southern University of Science and Technology, Shenzhen, 518055 China; 3 School of Materials and Environmental Engineering, Chengdu Technological University, Chengdu, 611730 China; 4 School of Materials and Energy, University of Electronic Science and Technology of China, Chengdu, 611731 China; 5 School of Mechanical and Electrical Engineering, University of Electronic Science and Technology of China, Chengdu, 611731 China

**Keywords:** Solid-state electrolyte, Polyethylene oxide, Grain boundary, Reforming, Li-ion battery

## Abstract

Polyethylene oxide (PEO)-based solid polymer electrolyte (SPE) is considered to have great application prospects in all-solid-state li-ion batteries. However, the application of PEO-based SPEs is hindered by the relatively low ionic conductivity, which strongly depends on its crystallinity and density of grain boundaries. In this work, a simple and effective press-rolling method is applied to reduce the crystallinity of PEO-based SPEs for the first time. With the rolled PEO-based SPE, the LiFePO_4_/SPE/Li all-solid li-ion battery delivers a superior rechargeable specific capacity of 162.6 mAh g^−1^ with a discharge-charge voltage gap of 60 mV at a current density of 0.2 *C* with a much lower capacity decay rate. The improvement of electrochemical properties can be attributed to the press-rolling method, leading to a doubling conductivity and reduced activation energy compared with that of electrolyte prepared by traditional cast method. The present work provides an effective and easy-to-use grain reforming method for SPE, worthy of future application.

## Introduction

Due to the high energy density and excellent safety performance, solid-state li-ion batteries are extensively regarded as promising systems for next-generation rechargeable electrochemical energy storage [1–4]. To achieve high performance all-solid-state li-ion battery, solid-state electrolytes should have satisfactory high ionic conductivity, good mechanical/electrochemical stability, and adequate electrode-electrolyte interface [2–4].

Polyethylene oxide (PEO)-based solid polymer electrolyte (SPE) has great application prospects due to its good flexibility, good compatibility of lithium metals, easy process, and low cost [5]. In addition to solid li-ion battery, PEO-based SPE also has a wide application prospect in many fields such as Mg-ion battery and Li-S battery [6–8]. However, the low conductivity greatly hindered the application of PEO-based SPEs: the PEO electrolytes exhibit a conductivity which ranges from 10^−8^ to 10^−6^ S cm^−1^ at room temperature, and the low conductivity will increase the battery internal polarization, and decrease the discharge-charge capacity and energy efficiency [9–12]. In PEO-based SPEs, li-ion forms a coordination bond with oxygen in PEO and migrates through continuous coordination and dissociation with oxygen atoms. Therefore, the mobility of li-ion mainly depends on the movement of polymer chain segments at the grain boundary and amorphous phase region, and the ion conductivity through the grain boundary and amorphous phase region is much higher than that through the crystalline lamellae [10].

To decrease the crystallinity of PEO and improve the conductivity of SPEs, different approaches have been developed and applied, such as filling and grafting. Nano-sized fillers have been widely used in PEO-based SPE, including nano-sized Al_2_O_3_, TiO_2_, SiO_2_, Li_0.3_3La_0.557_TiO_3_, and Li_6.4_La_3_Zr_1.4_Ta_0.6_O_12_ [12–17]. These nano-sized fillers can inhibit the PEO crystallization and promote the formation of grain boundaries and amorphous regions. Besides, some high ionic conductivity fillers can also provide additional ion transport pathway for li-ion transport [13–15]. Grafting also reduces the crystallinity of PEO-based SPE. As an example, PEO has been grafted on a poly(hydroxylstyrene) backbone as well as block copolymers with polystyrene. The achieved macromolecular greatly suppresses the propensity of PEO chains for complex crystal formation and thus improve the ionic conductivity of SEPs [18].

Press rolling is a normal reforming technique for metal processing [19–21]. By applying an external force on the metal surface, press rolling can crush and refine the grains, as well as increase the grain boundaries’ proportion and the hardness of the metal [22, 23]. Because of its simple process, low cost, high efficiency, and obvious grain refinement effect, press-rolling method is widely used for fabricating large bulk sheet or plate samples. Because press rolling can break grains and increase grain boundaries and amorphous phase, it has the potential to be applied to PEO-based SPEs to reduce the crystallinity of electrolytes and enhance conductivity. In this work, we report a simple and facile press-rolling route to prepare a PEO-based solid-state electrolyte with high ionic conductivity for solid-state li-ion batteries. This new method has the following features: (i) after rolling treatment, the spherulites of polymer electrolyte are crushed and reformed, resulting in a decrease in crystallinity and a double increase in conductivity and (ii) with the low crystallinity, PEO-based SPEs can provide more transport pathway for li-ion to equilibrium current distribution on lithium surface to prevent dendrite growth. Besides, the press-rolling method to form the PEO-based SPEs proposed in this work is very straightforward.

## Method and Characterization

### Preparation of Solid Polymer Electrolyte

Analytical grade chemicals polyethylene oxide (PEO, Mw = 600,000), nano-sized aluminum oxide (Al_2_O_3_, *d* ≤ 20 nm), bis(trifluoromethane)sulfonimide lithium salt (LiTFSI), and acetonitrile are purchased from Aladdin, China, and used as received.

The original solid polymer electrolyte was prepared by a simple cast method: the PEO, Al_2_O_3,_ and LiTFSI were mixed into acetonitrile for 24 h with EO/Li molecular ratio of 16/1 and PEO/Al_2_O_3_ weight ratio of 90/10, and then the obtained white suspension was cast into a polytetrafluoroethylene mold and dried in dry N_2_ flow at room temperature for 24 h. The resulting transparent electrolyte is named as PAL-C and then transferred to a dryer for preservation. To prepare the PAL-R electrolyte, an as-prepared PAL-C solid polymer electrolyte was cold-rolled in a roller press under the line loading of 150 N mm^−1^. In order to eliminate the influence of electrolyte thickness on performance, the thicknesses of each electrolyte were controlled to be ~ 135 μm.

### Electrode Preparation

The positive electrode was prepared through the conventional doctor-blade method with the LiFePO_4_ (LFP, BTR New Energy Material Ltd., China), Acetylene black (AB), PEO, and LiTFSI mass ratio of 7:1:1.4:0.6. The PEO and LiTFSI were firstly dissolved completely in acetonitrile and then the LFP and AB were added to the obtained transparent solution. A uniform slurry was got after magnetic stirring the mixture for 24 h then coated on aluminum foil through the conventional doctor-blade method. The electrode was then dried at 80 °C for 12 h and was finally cut into circular disks with the diameter of 12 mm. The active material mass loading of the as-prepared LFP electrode is controlled to be ~ 1.5 mg cm^−2^.

### Electrolyte Characterizations

The grain morphologies of the PAL-C and PAL-R electrolyte were obtained by scanning electron microscopy (SEM, JEOL-7500F). The crystallinity was analyzed with an X-ray diffraction system (XRD, model PW1825) using a Cu-Kα source operating at 40 keV. Differential scanning calorimetry (DSC) measurements were tested on a TA Instrument (Q5000IR) with the heating rate of 5 °C min-1 from − 70 to 10 °C under N_2_ atmosphere. The ratio of stress to strain was evaluated by the stress-strain curves and the tensile strength was taken on tensile-testing machine (CMT6104, China) as the stress value at the maximum of the curves.

### Electrochemical Performance of PEO-Based Polymer Solid Electrolyte

The as-synthesized polymer solid electrolyte was cut into a disk with a diameter of 16.5 mm for electrochemical performance test. The ionic conductivities of solid polymer electrolytes were measured in CR2032 cells by sandwiching the solid electrolyte between two polished stainless steel (SS) sheets (*d* = 14.0 mm). The ionic conductivity was obtained on SS/SPE/SS cell by electrochemical impedance spectroscopy (EIS) on CHI660E electrochemical station with frequency range from 1 to 100 mHz at temperature from 25 to 65 °C. The inhibitory effect of PEO-based SPE on lithium dendrite growth was conducted in the Li/SPE/Li symmetry cell on Neware testing system (Neware, China) under the discharge-charge current densities of 0.1, 0.2, and 0.3 mA cm^−2^ at 60 °C, respectively. The li-ion transference number (*t*_Li+_) of different electrolytes has been evaluated by a combination measurement of AC impedance and DC polarization using the method described by Evans et al. [21]. The polarization currents for a symmetric Li/SPE/Li cell (including the initial (*I*_o_) and steady-state (*I*_s_) values of current) under a small polarization potential (ΔV) at 10 mV were recorded. Meanwhile, the initial and steady-state values of the Li/electrolyte interfacial resistances (*R*_0_ and *R*_s_) were examined with impedance measurements before and after the DC polarization. The *t*_Li+_ was calculated with the Bruce-Vincent-Evans equation:

1 $$ {t}_{{\mathrm{Li}}^{+}}=\frac{I_{\mathrm{s}}\left(\Delta \mathrm{V}-{I}_0{R}_0\right)}{I_0\left(\Delta \mathrm{V}-{I}_{\mathrm{s}}{R}_{\mathrm{s}}\right)} $$

The battery performance of solid polymer electrolytes was tested in the all-solid-state li-ion battery with as-prepared LFP cathode, PEO-based SPE, and Li metal anode. The batteries were assembled in an argon-filled glovebox (DELLIX, China, water and oxygen ≤ 0.1 ppm) without any other liquid electrolyte. The galvanostatic discharge-charge tests were conducted on a battery cycling system at a current density of 0.1 *C* (1 *C* = 170 mA g^−1^) with voltage range from 2.0 to 3.75 V. Before the charge-discharge process, charge transfer resistances were measured by electrochemical impedance spectra (EIS) operated with the frequency range of 100 kHz to 0.1 Hz and an AC voltage amplitude of 5 mV. The rate and cycling performance were obtained from 0.1 *C* to 1 *C* and 0.5 *C*, respectively. All battery performance tests mentioned above were carried out at 60 °C on Neware testing system (Neware, China).

## Results and Discussion

**Fig. 1:**
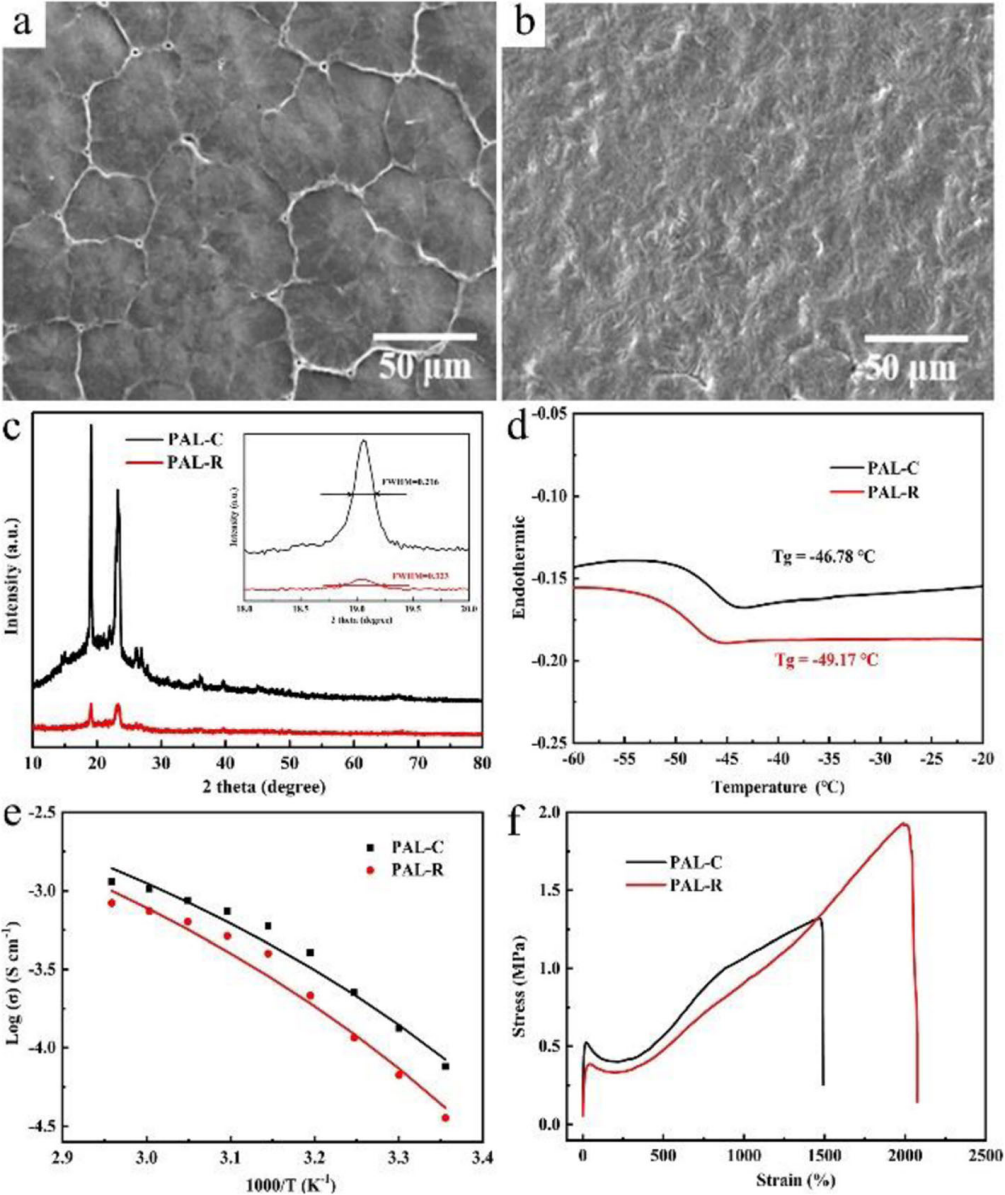
Material characterization of different SPES: **a**, **b** SEM images of PEO grain in SPE before (**a**) and after (**b**) press rolling; **c** XRD patterns for different SPE (inset: the enlarged XRD peaks at 19.0°); **d** DSC profiles; **e** ionic conductivity; and **f** stress-strain curves of PAL-C and PAL-R SPEs

The SEM images of the obtained electrolytes directly reveal the grain size and grain boundary distribution of as-prepared electrolyte (PAL-C) and rolled electrolyte (PAL-R) electrolyte. The PAL-C electrolyte exhibits a compact spherulite polycrystalline structure with the spherulite diameter of 50 μm (Fig. 1a and S1a). For PEO-based SPE, li-ions are thought to be mainly transported through grain boundaries and amorphous phase. Therefore, large-grained PAL-C SPE obtained by casting is unfavorable for li-ion transport and limits the conductivity of electrolytes. Rolling treatment can break the electrolyte grain, which can significantly reduce the crystallinity and increase li-ion transport pathway. After press rolling, the large spherulite disappeared and the electrolyte showed a relatively homogeneous structure for PAL-R (Fig. 1b and S1b). This uniform homogeneous structure is considered to have obvious advantages in improving the conductivity of SPEs.

In order to further analyze the change of crystallinity before and after rolled, XRD test was conducted and the results are shown in Fig. 1c. The diffraction peaks of PAL-C electrolyte at 19.0° and 23.2° are sharp and intense, indicating the highly crystalline nature [22, 23]. As a comparison, the diffraction pattern of PAL-R exhibits several broad and weak peaks, suggesting that the crystallinity of the PAL-R greatly reduced after press rolling. Besides, the main XRD peaks of PEO at 19.0° are also characterized by significant changes in the full width at half maximum (0.216 for PAL-C and 0.323 for PAL-R), implying that the amorphous phase in the electrolyte has increased. The decrease in crystallinity is believed to have a significant effect on the improvement of the conductivity.

The DSC profiles of PAL-C and PAL-R electrolytes were tested and shown in Fig. 1d, which reveals the glass transition temperature (*T*_g_) differences between the two electrolytes. The results suggest that the *T*_g_ of PAL-R is − 49.17 °C, which is lower than that of PAL-C (− 46.78 °C). This result shows that in PAL-R electrolyte, the movement of polymer segments can occur at lower temperature, which leads to a higher ionic conductivity than that in PAL-C electrolyte.

Ion conductivity σ of PAL-C and PAL-R SPE is calculated with the following equation:

2 $$ \sigma =L/ RS $$

where *S*, *L*, and *R* represent the geometric area of stainless steel blocking electrodes, the thickness of electrolytes, and the bulk resistance of the sample obtained from the impedance plots, respectively. The impedance spectra of PAL-C and PAL-R solid polymer electrolytes at different temperatures are tested and shown in Fig. S2. Figure 1 c shows the temperature dependence of the calculated ionic conductivity of the PAL-C and PAL-R electrolytes. The as-prepared PAL-R electrolyte reaches an ionic conductivity of 7.58 × 10^−5^ S cm^−1^ at 25 °C and 1.03 × 10^−3^ S cm^−1^ at 60 °C, which is two times higher than that of PAL-C electrolyte (3.58 × 10^−5^ S cm^−1^ at 25 °C and 7.43 × 10^−4^ S cm^−1^ at 60 °C) and better than that of PEO-based SPE prepared through other methods [14, 24, 25]. The enhancement in li-ion conductivity is attributed to the crystallinity reduction of the PEO-based SPE after the press-rolling process and is expected to lead to good battery performance. The relationship between log *σ* and 1000/T of the PAL-C and PAL-R SPEs reveals that the temperature dependence of conductivity follows Vogel-Tammann-Fulcher (VTF) empirical equation [10, 16, 26, 27]:

3 $$ \sigma ={\sigma}_0{T}^{-1/2}\exp \left(-{E}_a/ RT\right) $$

where *σ*, *E*_*a*_, *σ*_0_, *T*, and *R* represent the ionic conductivity, activation energy, pre-exponential factor, a temperature factor, and the ideal gas constant, respectively. The *E*_*a*_ of PAL-C and PAL-R was calculated using the VTF equation (Fig. 1e), and the results show the fitting value of *E*_*a*_ for PAL-R is 5.0 × 10^−2^ eV, which is much smaller than that for PAL-C (5.8 × 10^−2^ eV). The lower *E*_*a*_ demonstrates that the li-ion movement in PAL-R electrolyte needs less energy than that in PAL-C electrolytes, indicating a higher conductivity.

The mechanical property of SPE is directly related to its barrier effect to lithium dendrite. Figure 1 f shows the stress-strain test results of PAL-C and PAL-R SPEs. The ductility of the PAL-R SPE reaches to 1990%, which is much higher than that of PAL-C SPE (1470%). This reinforced ductility of PAL-R SPE would significantly improve the tolerance to dendrite penetration and inhibit short circuit in batteries. The electrochemical windows of two different solid polymer electrolytes have been tested with linear sweep voltammetry method and the results are shown in figure S2. The decomposition voltages of both electrolytes were tested to be as high as 5.8 V, suggesting that the stability of the solid polymer electrolyte has no change after the press rolling. The real densities of the two electrolytes are obtained by using Archimedes drainage method with kerosene as the medium, and the densities of PAL-C and PAL-R are calculated to be both 1.38 ± 0.02 g cm^−3^. The results show that the achieved conductivity difference of these two electrolytes comes from the grain boundary difference rather than the density change.

**Fig. 2:**
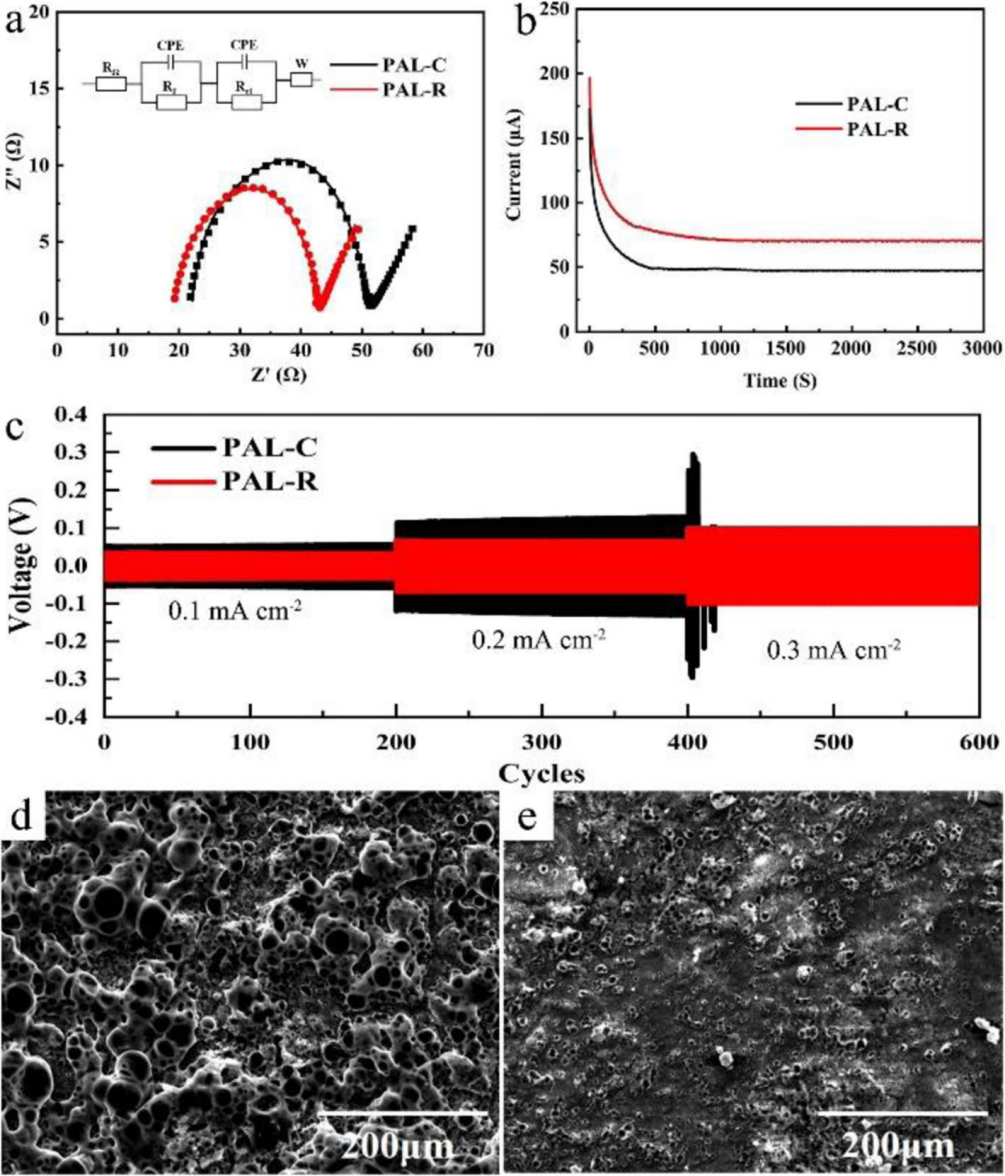
**a** EIS plots (inset: the equivalent circuit model). **b** DC polarization curve and **c** cycling performance of Li/SPE/Li symmetric cells with different SPEs. **d** and **e** The morphology of Li metal after 200 cycles in Li/SPE/Li symmetric cell with PAL-C and PAL-R SPE, respectively

The inhibiting effect of the two SPE on lithium dendrite grows is tested with a Li/SPE/Li symmetric cell. Before the test proceeds, electrochemical impedance spectroscopy (EIS) is conducted to analyze the Li-SPE interface properties of different cells and the results are shown in Fig. 2a. The EIS plots are fitted with a simple mode which consists of ohmic resistance (*R*_Ω_), interface resistance (*R*_f_), charge transfer resistance (*R*_ct_), constant phase elements (CPE1 and 2), and Warburg diffusion resistance (Wo) [28, 29]. The simulated results of *R*_Ω_, *R*_f_, and *R*_ct_ in the battery using PAL-R electrolyte are calculated to be 19.12, 5.72, and 17.65 Ω, respectively, which are smaller than those using PLA-C electrolytes (21.83, 5.99, and 21.77 Ω). The decreased solution resistance (*R*_Ω_) and interfacial resistance (*R*_f_) can be attributed to two reasons: (i) after rolling press, more grain boundaries are created in PAL-R electrolyte, leading to a higher conductivity and lower solution resistance. (ii) The relatively smooth electrolyte surface and increased grain boundaries are beneficial to improve the surface contact between electrolyte and lithium metal, leading to a lower solution resistance and interfacial resistance. The decreased solution resistances and improved interfacial connection provide more li-ion transport pathways and reaction interfaces for the electrochemical reaction, eventually leading to the reduction of the charge transfer resistance (*R*_ct_). After the initial EIS test, a 10-mV DC voltage was applied to the Li/SPE/Li symmetric cells to investigate the li-ion transference number in different SPEs [30, 31]. Based on the current-time curve (Fig. 2b), impedance before and after polarization (Fig. 2a and S3), the li-ion transference number for PAL-R SPE is calculated to be 0.24, which is higher than the value of PAL-C SPE (0.16). This improvement can be attributed to the reduction of crystallinity phase, which releases more li-ions for ion transportation. After the EIS test, the Li/PAL-C/Li and Li/PAL-R/Li symmetric cells were charged and discharged at 60 °C for 30 min under current densities of 0.1, 0.2, and 0.3 mA cm^−2^, respectively (Fig. 2c). From this result, we can find that the voltage of Li/PAL-R/Li cell can be stabilized at 33 mV and 67 mV at current densities of 0.1 and 0.2 mA cm^−2^, respectively, which are much smaller than that of Li/PAL-C/Li (56 and 126 mV). For higher current density (0.3 mA cm^−2^), PAL-R SEP can stably cycle for 200 cycles, but dendrite penetration occurs after only a few cycles of PAL-C SPE under the same current density. The surface morphologies of lithium electrode with different SPEs after 200 cycles at 0.2 mA cm^−2^ were tested and shown in Fig. 2c, d. There are massive irregular lithium dendrites with the PAL-C SPE but a relatively smooth lithium surface with PAL-R SPE could be found. This result can be attributed to the high ionic conductivity and uniform ion transportation pathway of PAL-R SPE, which will lead to the uniform lithium deposition to avoid the internal short circuit caused by lithium dendrite growth.

**Fig. 3:**
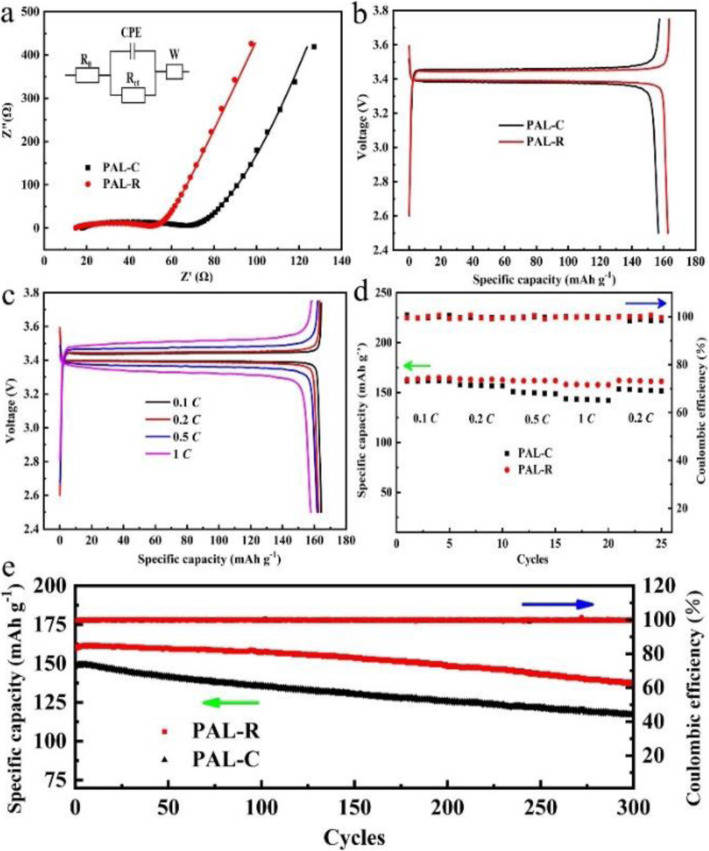
**a** EIS plots and simulated results for LFP/SPE/Li batteries with different SPEs (inset: the equivalent circuit model), **b** discharge-charge performances of LFP/SPE/Li batteries with different SPEs at 0.2 *C*, **c** discharge-charge curves of LFP/SPE/Li battery with PAL-R SPE at different current densities, **d** rate performance; and **e** long-cycle performance of LFP/SPE/Li batteries with different SPEs

Galvanostatic charge-discharge performance of all-solid li-ion batteries containing LiFePO_4_ (LFP) cathode, Li anode with different SPEs are tested and the results are shown in Fig. 3. Before galvanostatic charge-discharge test, the impedance of each battery was tested and fitted with an equivalent circuit model (inset of Fig. 3a). In this model, *R*_Ω_ corresponds to the ohmic resistance; *R*_ct_ represents the charge transfer resistance for electrochemical reactions; CPE is the constant phase angle element related to the double-layer capacitance of porous cathode, and *Z*_w_ is the finite length Warburg contribution. It is found that *R*_Ω_ decreases from 17.1 to 14.4 Ω and *R*_ct_ decreases from 47.5 to 33.1 Ω for battery with PAL-C and PAL-R SPE, respectively, as shown in Fig. 3a. The decreased *R*_Ω_ and *R*_ct_ can be attributed to the lower crystallinity of PAL-R SPE, which can provide more li-ion transportation pathways to enhance the conductivity of the electrolyte and facilitate redox reaction in the LFP electrode simultaneously. Figure 3 b shows the charge-discharge capacities of all-solid li-ion batteries with different SPEs at 60 °C under current density of 0.2 *C*. The battery with PAL-R electrolyte delivers a discharge capacity of 162.6 mAh g^−1^ with the discharge-charge voltage gap of 60 mV, while the battery with PAL-C electrolyte delivers a discharge capacity of 156.7 mAh g^−1^ with the discharge-charge voltage gap of 82 mV. The increased discharge capacity and decreased voltage gap can be attributed to the higher conductivity and lower resistances of the PAL-R electrolyte compared with PAL-C electrolyte. The rate performance of all-solid li-ion batteries with different SPE was conducted under current densities of 0.1 *C*, 0.2 *C*, 0.5 *C*, 1 *C*, and 0.2 *C* (Fig. 3c, d and S4), respectively. The results indicate that the battery with PAL-R can deliver a capacity of 164.3, 162.6, 161.8, 157.8, and 161.2 mAh g^−1^, respectively. This performance is much better than the battery with PAL-C electrolyte, which only delivers capacities of 161.5, 156.7, 148.7, 142.1, and 151.8 mAh g^−1^, respectively. This result illustrated that the PAL-R electrolyte could afford the high-rate operation due to the higher conductivity.

The cycle performance of the battery with different SPE was tested under the current density of 0.5 *C* (Fig. 3e). For LFP/PAL-C/Li cell, the discharge capacity maintains 117.1 mAh g^−1^ after 300 cycles with the capacity decay rate of 0.071% per cycle. For comparison, the LFP/PAL-R/Li cell maintains a discharge capacity of 136.8 mAh g^−1^ with the capacity decay rate of 0.048% per cycle under the same condition. In the existing all-solid-state LFP/PEO-SPE/Li li-ion battery, the capacity decay mainly comes from two aspects: (i) the continuous generation and growth of lithium dendrites on anode dilute the contact between Li electrode and electrolyte, resulting in the increased anode resistance. (ii) Although PEO has good stability, it still decomposes during the charge-discharge voltage range [14, 29]. The accumulated decomposition products will gradually increase the reaction resistance of the cathode electrode. In all-solid battery with PAL-R electrolyte, the increase of anode resistance is inhibited because of good dendrite inhibition performance. However, the continuous decomposition reaction in cathode electrode causes increasing battery resistance and gradually becomes the main reason of battery capacity decay after 100 cycles. As a contrast, all-solid battery with PAL-C electrolyte surfers from both the increasing anode resistance and cathode resistance, leading to a continuous capacity decay during 300 cycles. The results show that the rolling treatment can improve the electrochemical performance of the electrolyte and a more stable solid state electrolyte should be developed for future application.

## Conclusion

In this work, we applied a simple press-rolling technology to improve the performance of PEO-based SPE for all-solid li-ion batteries. The rolled PEO-based SPE shows a decreased crystallinity and increased amorphous phase, which are expected to be benefit for li-ion transportation. After treatment, PEO-based SPE delivers a doubling room temperature conductivity and decreased activation energy. It is experimentally shown the LiFePO_4_/SPE/Li all-solid li-ion battery with the rolled PEO-based SPE exhibits a rechargeable specific capacity of 162.6 mAh g^−1^ with a discharge-charge voltage gap of 60 mV at a current density of 0.2 *C*, which is much better than that of the cast PEO-based SPE (156.7 mAh g^−1^ and 82 mV). Furthermore, the capacity decay rate was reduced to 0.0048% per cycle after 300 cycles at 0.5 *C*. All the results show that the grain reforming technology is a promising technology to improve the performance of PEO-based SPE.

## Additional File

**Additional file 1: Fig.S1.** SEM images of PEO grain in SPE before (a) and after (b) press rolling. **Fig.S2.** electrochemical window test of (a) PAL-R and (b) PAL-C electrolyte. **Fig.S3.** EIS plots of SS/SPE/SS cell with PAL-C (a) and PAL-R (b) electrolyte under different temperatures. **Fig.S4.** EIS plots of Li/SPE/Li symmetric cells with different SPEs after DC polarization.

## Data Availability

All data generated or analyzed during this study are included in this published article and its supplementary information files.

## Abbreviations

PEO: Polyethylene oxide
LFP: LiFePO_4_
SPE: Solid polymer electrolyte
AB: Acetylene black
LiTFSI: Bis(trifluoromethane)sulfonimide lithium salt
SEM: Scanning electron microscopy
XRD: X-ray diffraction
DSC: Differential scanning calorimetry
EIS: Electrochemical impedance spectroscopy
SS: Stainless steel
