# Supplementary material for: Improving the Conductivity of Solid Polymer Electrolyte by Grain Reforming
Source: Nanoscale Res Lett. 2020 May 26;15:122. doi: 10.1186/s11671-020-03355-4 (PMC7251041; doi:10.1186/s11671-020-03355-4)
Supplement: Supplementary file 1 — Additional file 1: Fig.S1. SEM images of PEO grain in SPE before (a) and after (b) press rolling. Fig.S2. electrochemical window test of (a) PAL-R and (b) PAL-C electrolyte. Fig.S3. EIS plots of SS/SPE/SS cell with PAL-C (a) and PAL-R (b) electrolyte under different temperatures. Fig.S4. EIS plots of Li/SPE/Li symmetric cells with different SPEs after DC polarization. [file 11671_2020_3355_MOESM1_ESM.docx]

Improving the conductivity of solid polymer electrolyte by grain reforming

Zhaohuan Wei ^1, 2,*^, Yaqi Ren ^3^, Minkang Wang ^4,^ Weirong Huo ^4^, Hui Tang ^4,*^

^
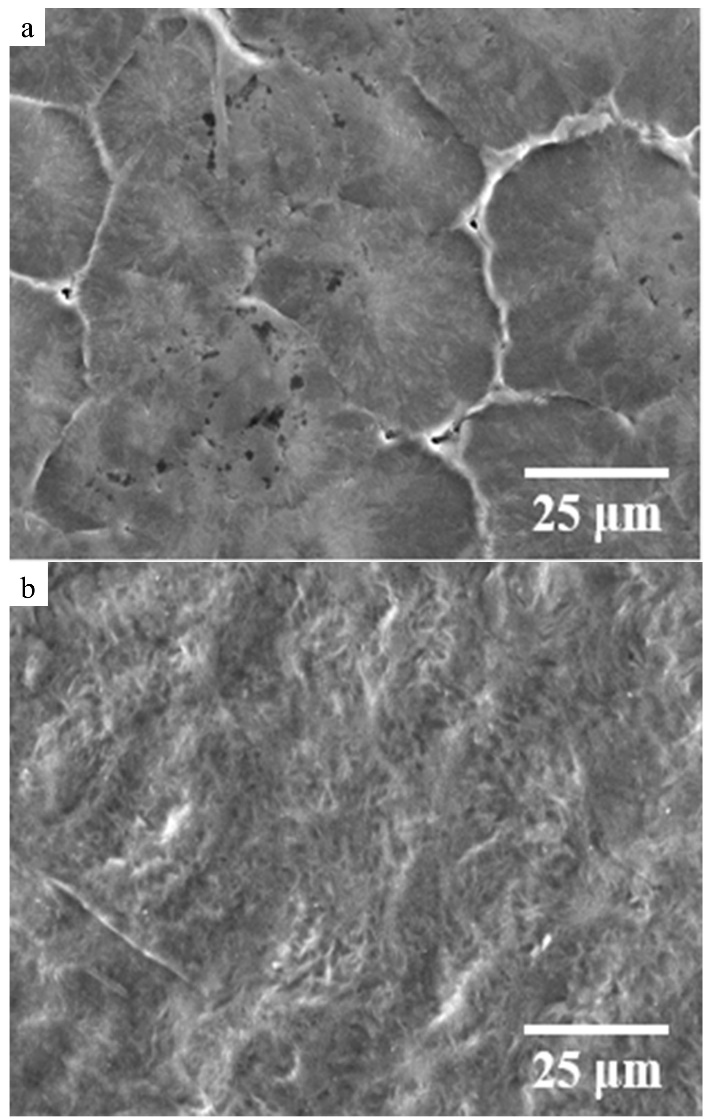
^

Fig.S1. SEM images of PEO grain in SPE before (a) and after (b) press rolling.


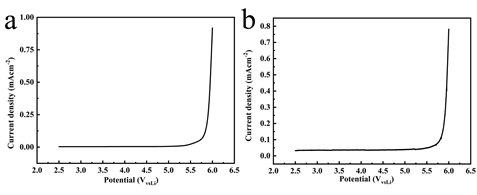


Fig.S2. electrochemical window test of (a) PAL-R and (b) PAL-C electrolyte.


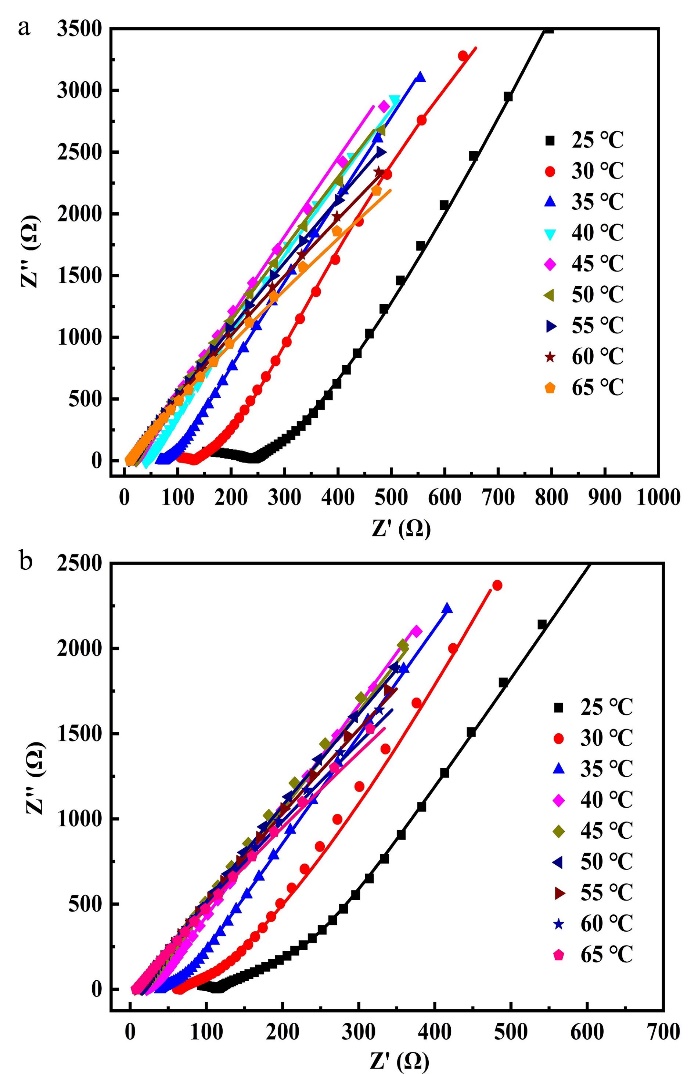


Fig.S3. EIS plots of SS/SPE/SS cell with PAL-C (a) and PAL-R (b) electrolyte under different temperatures.


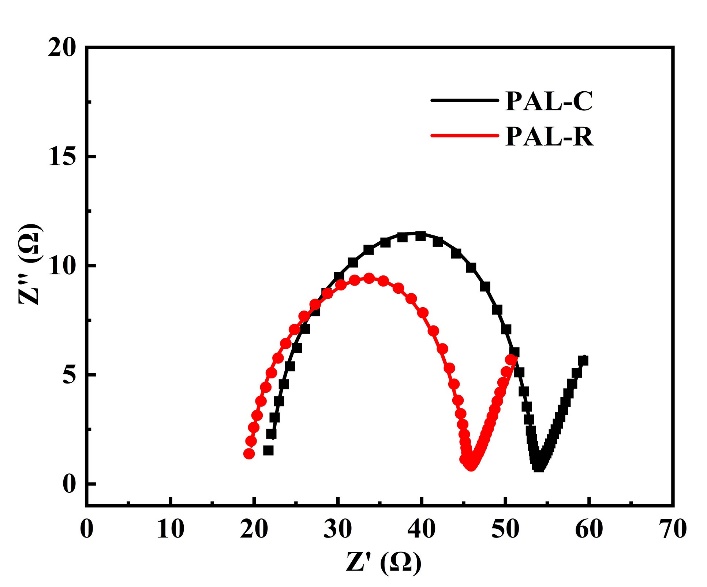


Fig.S4. EIS plots of Li/SPE/Li symmetric cells with different SPEs after DC polarization.


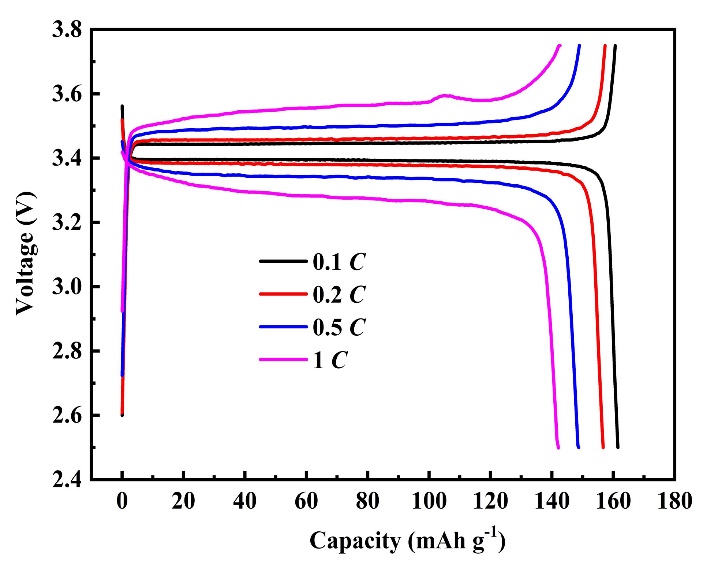


Fig.S5. Discharge-charge curves of LFP/SPE/Li battery with PAL-C SPE at different current densities.
